# Amygdalar Pattern Similarity to Negative and Neutral Images, Ratings of the Images, and PET-Measured Amyloid and Tau Levels in Older Adults Without Dementia

**DOI:** 10.1007/s42761-026-00394-5

**Published:** 2026-08-11

**Authors:** Mingtong Liu, Lauren K. Gresham, Nikki A. Puccetti, Tobey J. Betthauser, Sterling C. Johnson, Stacey M. Schaefer

**Affiliations:** 1 Institute on Aging, University of Wisconsin-Madison, Madison, WI 53706 USA; 2 The Ohio State University, Columbus, OH 43210 USA; 3 Wisconsin Alzheimer’s Disease Research Center, University of Wisconsin School of Medicine and Public Health, MC 2420, 600 Highland Avenue, Madison, WI 53792-2420 USA; 4 Department of Medicine, University of Wisconsin-Madison School of Medicine and Public Health, 1685 Highland Avenue, 5158 Medical Foundation Centennial Building, Madison, WI 53705-2281 USA

**Keywords:** Emotional reactivity, Amygdala, Alzheimer’s disease, Tau, fMRI, Representational similarity analysis

## Abstract

This study examines associations between brain levels of amyloid-β and tau with representational pattern similarity in amygdalar reactivity to negative and neutral images in older adults without dementia. 81 participants viewed affective images during functional magnetic resonance imaging (fMRI). Participants rated the images on valence and arousal outside the scanner. Amyloid-β and tau were measured with ^11^C-Pittsburgh compound B and ^18^F-MK-6240 positron emission tomography (PET) imaging. Representational pattern similarity analyses compared amygdalar responses to negative and neutral stimuli, while preserving the voxel-wise pattern of fMRI activation. Greater differentiation in the pattern of responding across the amygdala was indicated by lower similarity between negative and neutral stimuli. Greater tau levels in the entorhinal cortex and amygdalae were associated with less pattern similarity in left amygdalar reactivity to negative and neutral images in participants whose tau level was below standard positivity thresholds. Less pattern similarity in right amygdalar reactivity to negative and neutral images was also associated with the participants’ valence and arousal ratings of the stimuli. No associations were found with global amyloid levels and only the association between entorhinal tau and amygdalar similarity remained significant after Bonferroni correction. Greater amygdalar pattern separation in response to negative and neutral stimuli with higher levels of entorhinal tau suggest greater amygdalar sensitivity to negative compared to neutral information as tau accumulates in the brain, suggesting a potential underlying mechanism for the disrupted emotional processes often observed in preclinical Alzheimer’s Disease and other Related Dementias.

**Supplementary Information:**

The online version contains supplementary material available at https://doi.org/10.1007/s42761-026-00394-5.

Emotional abnormalities have been observed in the preclinical stage of Alzheimer’s disease (AD) and Related Dementias (ADRD), including higher levels of depressive (Berger et al., 1999) and anxiety symptoms (Hanseeuw et al., 2020). Anxiety, depression, and apathy often precede the onset of AD (Johansson et al., 2020, 2022; Masters et al., 2015). Cognitively normal older adults, who later were found to be amyloid positive, self-reported differences in their emotional reactivity on a neuroticism composite assessing anxiety, depression, and vulnerability in response to socially salient stimuli (Fredericks et al., 2018). Emotional processing deficits can also manifest early in the progression to AD, with the impaired identification of negative emotions in facial expressions being frequently reported in AD (Chaudhary et al., 2022). Other neurodegenerative disorders also exhibit disturbances in emotional processing, such as behavioral variant frontotemporal dementia (bvFTD), which is often characterized by apathy, emotional blunting, and difficulties recognizing emotion in others (Hodges & Piguet, 2018). Higher levels of mental health symptomatology have also been reported in other forms of dementia. For example, anxiety is observed frequently in Lewy body dementia occurring up to 4–5 years before the diagnosis (Segers et al., 2020).

These emotional abnormalities may be related to pathological proteins that accumulate during the development of ADRD. Higher baseline levels of amyloid-β pathology, a key hallmark of AD (Sadigh-Eteghad et al., 2015; Sehar et al., 2022), were associated with greater increases in apathy and anxiety in cognitively unimpaired older adults (Johansson et al., 2022). The neuroticism composite measure assessing anxiety, depression, and vulnerability in emotional responses increased with age in people who became amyloid-positive (Fredericks et al., 2018). Greater depressive symptoms have also been associated with greater tau burden level in the amygdala in cognitively unimpaired individuals at higher risk for developing AD due to the E280A mutation on the Presenilin-1 gene (Tristão-Pereira et al., 2025). Collectively, these studies support a linkage between impaired emotional processes with amyloid-β and tau burden.

Emotional reactivity to affective stimuli can be measured with neuroimaging to better understand the neural basis of emotional dysfunction. This study examined whether neural reactivity to negative compared to neutral stimuli measured by functional magnetic resonance imaging (fMRI) was associated with amyloid-β and tau levels. An amygdala region of interest was chosen due to the amygdala’s essential role in emotional processing (Anders et al., 2008; Janak & Tye, 2015; Mather et al., 2004; Preckel et al., 2019). Negative emotional reactivity was represented by amygdala pattern similarity in response to negative compared to neutral images using representational similarity analysis (RSA; Kriegeskorte, 2008), with less similarity indicating greater differentiation in the amygdala’s pattern of reactivity to negative and neutral stimuli (Hu et al., 2022). RSA approaches take advantage of the multivariate nature of fMRI data rather than the aggregated response in univariate analysis (Popal et al., 2019). In other words, RSA captures fine-grained, spatial activity patterns which include critical and detailed information about properties of stimuli compared to univariate analyses which average activity across all voxels in a brain region of interest. RSA approaches are useful for measuring individual differences in emotional processes in the brain and have previously been used to investigate the neural underpinnings of emotional similarity (Riberto et al., 2022), brain similarity during emotional perception and later memory (Park et al., 2021), and lingering negative emotional processes by examining amygdala representational similarity between negative images followed by neutral faces (Puccetti et al., 2021).

In this study, associations were investigated between individual differences in the representational similarity in response to affective images in the amygdala with both the image valence and arousal ratings and PET-measured amyloid-β and tau levels in the brain. Specifically, this study examined associations between amygdalar response pattern similarity to negative and neutral images with (i) participants’ valence and arousal ratings of the images (ii) global amyloid-β PiB binding; (iii) regional tau burden in the bilateral entorhinal cortex and amygdalae, and (iv) as a function of amyloid-β (A+/-) or tau positivity (T+/-) according to standard positivity thresholds to better understand how accumulating pathology is associated with emotional responding compared to a clinical elevated level of pathology in the brain. Standard positivity thresholds refer to the established criteria to classify individuals along the AD continuum as reaching sufficient levels to be positive or negative for amyloid and tau using PET biomarkers (Betthauser et al., 2019; Johnson et al., 2014).

## Method

### Participants

Data were obtained from 91 participants from The Wisconsin Registry for Alzheimer’s Prevention (WRAP; Johnson et al., 2018), a longitudinal study investigating factors that influence the risk of developing AD. Participants did not show symptoms of dementia (see below) and did not have any significant neurologic disease or cognitive impairment at the time of enrollment. Neither structural MRI nor PET markers were used for participant selection. Ten participants were excluded from analyses because 1 participant did not complete the fMRI scan, 2 did not respond behaviorally with button presses during the fMRI task, and 7 had excessive motion in 2 or more fMRI runs. In sum, 81 participants who completed task-based fMRI, valence and arousal ratings of the images, and PET imaging were included in our final analyses (mean age: 66.56 ± 6.34 (*SD*) years, age range: 51–78 years, 56 Females, 5 Black/African American; see Table 1).

**Table 1:** Demographic Characteristics of Participants

Participant Characteristics	All	Amyloid-Negative (A-)	Amyloid-Positive (A+)		Tau-Negative (T-)	Tau-Positive (T+)
Sample Size	81	68		13	74	7
Mean Age (years)	66.56	66.06		69.15	66.14	71.00
% Female	69%	71%		62%	68%	86%
% Black/African American	6%	6%		8%	7%	0

According to previously established criteria using PET biomarkers, the threshold of A + was set as global amyloid-β distribution volume ratio (cerebellum gray matter reference region) > 1.19 (Betthauser et al., 2020; Racine et al., 2016), and the threshold of T + was set as bilateral entorhinal MK-6240 standard uptake value ratio (inferior cerebellar gray matter reference region, 70–90 min post-injection) > 1.27 (Betthauser et al., 2020). These thresholds were based on agreement with visual rating and Gaussian Mixture Modeling, respectively

Inclusion criteria to the WRAP include not exhibiting dementia criteria at enrollment and having good general health with no conditions/medications affecting cognition or imaging (see Supplementary material). Participants who do not have the cognitive competence and legal capacity to make informed medical decisions are excluded at entry. As part of their WRAP participations, participants completed a neuropsychological battery at their first visit, 2 or 4 years later for their second visit, and approximately every 2 years following (see Johnson et al., 2018 for full details about the WRAP sample and longitudinal data collection). In addition, participants receive a research diagnosis after each visit. Briefly, a team of clinicians (physician dementia specialists, nurse practitioners, and neuropsychologists) blind to biomarker data make research diagnoses. Those who met criteria for impairment reflecting a change from a prior normal level of function are assigned a research diagnosis of mild cognitive impairment when applicable. Importantly, no participants included in the analyses in this paper received a diagnosis of dementia prior to their PET or fMRI.

Informed written consent was obtained from all participants before they participated in this study. This study was approved by the University of Wisconsin-Madison’s Health Sciences Institutional Review Board (IRB) before data collection and was conducted in accordance with the ethical standards in the 1964 Declaration of Helsinki and its later amendments.

### PET Imaging

Participants completed ^18^F-MK-6240 and ^11^C-Pittsburgh compound B (PiB) PET imaging and structural MRI imaging (Betthauser et al., 2020; Johnson et al., 2014). PET imaging and structural MRI data were reconstructed, processed, and quantified using SPM12 and Matlab (as previously described in detail Betthauser et al., 2019; Johnson et al., 2014). Bias correction, tissue class segmentation, and spatial normalization were performed on structural MRI data. Inverse warping was performed to transform regions of interest in MNI152 template to subject space. Regions of interest were then restricted to grey matter probabilities > 0.3, smoothed with a 6 mm Gaussian kernel, and defined by retaining voxels > 0.7. Briefly, regional binding estimates (standard uptake value ratio, SUVR; distribution volume ratio, DVR) were extracted from MRI-guided pipelines. Several participants completed longitudinal PiB and MK-6240 PET scans, so data from PiB and MK-6240 PET scans closest to the task-based fMRI scan were used in our analyses (fMRI 0.08 ± 0.65 years after PiB, and 0.06 ± 0.63 years after MK-6240).

Amyloid-β and tau levels and positivity status were indexed by *global* amyloid-β and *entorhinal cortex* tau burden based on previously established PET criteria in the literature and in the WRAP sample (Betthauser et al., 2020). Amyloid deposition is diffusely distributed and is commonly quantified using global cortical mean tracer uptake. In contrast, tau accumulation shows a more hierarchical pattern of spread, and therefore the entorhinal cortex is commonly used to quantify tau tracer update (e.g., Weigand et al., 2022). These accepted practices for defining amyloid vs. tau positivity are how positivity has been defined in the Wisconsin Registry for Alzheimer’s Prevention (Betthauser et al., 2020), from which our participants were recruited. The global cortical PiB DVR average was calculated for continuous analyses and used to dichotomize individuals as amyloid positive or negative using a previously defined global DVR threshold of 1.19 (Betthauser et al., 2020; Racine et al., 2016). In comparison, Betthauser and colleagues established the entorhinal cortex as the region of interest for MK-6240 SUVRs based on the entorhinal cortex being the first region involved in pathological staging of neurofibrillary tangles (Betthauser et al., 2020; Braak & Braak, 1991). Moreover, Betthauser and colleagues found that the proportion of variance in MK-6240 SUVR explained by both global PiB DVR and age was highest in the entorhinal cortex and decreased for composite regions associated with later Braak stages, thereby supporting their use of the entorhinal ROI to classify tau positivity, and our continued use of the entorhinal ROI. Therefore, the threshold of A + was set as global amyloid-β distribution volume ratio (cerebellum gray matter reference region) > 1.19 (Betthauser et al., 2020; Racine et al., 2016); while the threshold of T + was set as bilateral entorhinal MK-6240 standard uptake value ratio (inferior cerebellar gray matter reference region, 70–90 min post-injection) > 1.27 (Betthauser et al., 2020). As described above, the choice of ROIs for amyloid and tau PET imaging reflects the expected spatial distribution of the pathologies detected by PET and in preclinical sporadic Alzheimer’s disease. Given the cognitively unimpaired status of the participants, it is unlikely that we would observe much if any elevated tau PET binding outside of the mesial temporal lobe, which is typically the first region to detect tau pathology with current PET imaging tracers. In contrast to tau PET, amyloid PET becomes elevated across several brain regions nearly at the same time and there is less spatial specificity of this distribution. Therefore, the entorhinal cortex and the amygdala were selected as ROIs for tau PET imaging and the global amyloid was selected for amyloid PET imaging.

### fMRI Stimuli

Thirty negative, thirty neutral, and thirty positive images were presented during the fMRI scan, selected from the International Affective Picture System (IAPS; Lang et al., 2008) based on the normative ratings accompanying the images. Image characteristics such as luminosity, complexity, and social content were matched across valence sets. According to the normative ratings, rated valence of positive images was higher than neutral images (positive: 7.11 ± 0.50; neutral: 4.98 ± 0.23), and rated valence of neutral images was higher than negative images (negative: 2.96 ± 0.48). Negative and positive images were selected such that they were rated as equally arousing on average (negative: 5.46 ± 0.66; positive: 5.47 ± 0.53) and were both higher than that of neutral images (3.16 ± 0.42; *ts* > 16.1, *Ps* < 0.001). Forty-five faces with neutral expressions, consisting of approximately half female and half male, were gathered from the XM2VTSDB multi-modal face database (Messer et al., 1999), the Montreal Set of Facial Displays of Emotion (Beaupré & Hess, 2005), and the NimStim database (Tottenham et al., 2009). Grayscale images of neutral faces were cropped above the hair and below the chin, and glasses and facial hair were removed.

### Experimental Design

**Fig. 1:**
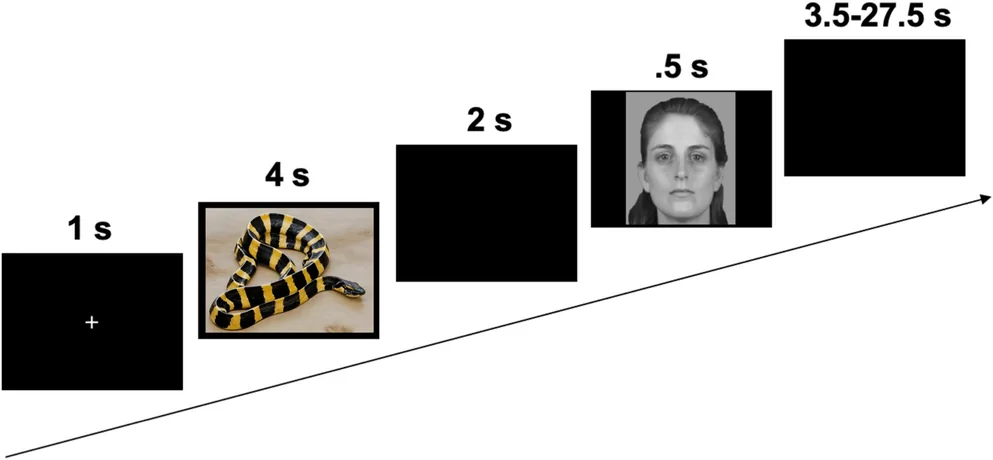
fMRI Trial Structure: On each trial, participants viewed either a negative, neutral, or positive image from the International Affective Picture System for 4 s. Following a 2 s delay, a neutral face was presented, and participants pressed a button to indicate the gender of the face. A total of 90 trials with 30 negative, 30 neutral, and 30 positive images were shown in a quasi-random order. The negative and neutral image presentations are the focus of this project

The fMRI scan consisted of 3 runs with 30 trials/run. In each run, participants viewed 10 negative, 10 neutral, and 10 positive images from the IAPS in a pseudorandom order. Each image was presented for 4 s after a white fixation cross displayed for 1 s (see Fig. 1 for the trial structure). Negative images included images of threatening animals, drug injection, guns, injury, dirty objects, etc.; neutral images included farm animals, calm people, street views, mushrooms, abstract art, etc.; and positive images included cute animals, smiling faces, flowers, athletes, nature scenes, etc. Following each image, a neutral face was displayed 2 s after image offset for 500 ms. A varied inter-trial interval of 3.5 s to 27.5 s, with an average of 7.5 s, was presented before each subsequent trial. Each neutral face was presented twice across the scan session, always after images of the same valence. To ensure that participants’ attention was focused on the task, they were instructed to identify the gender of the face by pressing one of two buttons on a button box.

After the fMRI scan and outside the scanner, participants were asked to rate the valence and arousal of the images viewed during the fMRI task. Participants were instructed to rate each picture “as you actually feel while you watch the picture.” After reviewing each image, participants separately rated the valence and arousal of the image on a scale of 1–9 using Bradley and Lang’s Self-Assessment Manikin (Bradley & Lang, 1994). In the valence scale, 1 represents unhappy and 9 represents happy. In the arousal scale, 1 represents calm and 9 represents excited.

### Imaging Protocol and Data Analysis

MRI data were acquired on a 3 Tesla scanner (MR750 GE Healthcare, Waukesha, WI) with a 32-channel head coil. Three sets of functional echoplanar images were obtained using a gradient echo sequence (EPIs; 231 volumes; TR = 2000 ms; TE = 20 ms; flip angle = 60°; field of view = 220 mm; slice thickness = 3 mm; slice spacing = 1 mm). Structural images were obtained using a three-dimensional magnetization-prepared T1-weighted rapid gradient echo (BRAVO) sequence (Mugler & Brookeman, 1990; 256 ⋅ 256 in-plane resolution; 160 axial slices; TR = 8.2 ms; TE = 3.2 ms, flip angle = 12°; field of view = 256 mm; slice thickness = 1 mm).

Structural and functional imaging data were preprocessed using ANTs (Advanced Normalization Tools v.2.4.3, https://github.com/ANTsX/ANTs) and FSL (FMRIB Software Library v.6.0.4, https://fsl.fmrib.ox.ac.uk/fsl) software packages. Skull stripping, which removes the non-brain tissue in T1-weighted structural images, was performed using ANTs. The fslreorient2std function was used to guarantee a consistent orientation of images. To reduce the influence of the non-steady state of the MR signal at the beginning of each run, the first four volumes from the task-based fMRI Blood Oxygenation Level Dependent (BOLD) sequence were excluded. Then, fsl_motion_outliers function was used to identify volumes that exceeded a 0.5 mm threshold of framewise displacement. Runs with more than 25% of volumes that exceed this threshold were removed from analysis. The following fMRI analysis was carried out using FEAT (FMRI Expert Analysis Tool v.6.00) package within FSL. Motion correction was performed using FSL’s MCFLIRT. Spatial smoothing was performed using a Gaussian kernel of full-width half maximum (FWHM) 5 mm. A high-pass filter with a cutoff at 100 s was used to remove low frequency drifts. Functional images were registered to both the structural images and a 2-mm resolution standard template in Montreal Neurological Institute (MNI) coordinate space. The pre-processed data were analyzed using a general linear model (GLM) as implemented in FEAT. Movement parameters obtained through pre-processing steps and response/no response time to face presentations were included within the multiple regression as covariates of no interest. Six explanatory variables (EVs) of interest were set for each participant at the first-level analysis, consisting of negative images, neutral images, positive images, faces following negative images, faces following neutral images, and faces following positive images. A contrast parameter estimates (COPE) image was generated for each of the six EVs for each participant.

### Representational Similarity Analysis

**Fig. 2:**
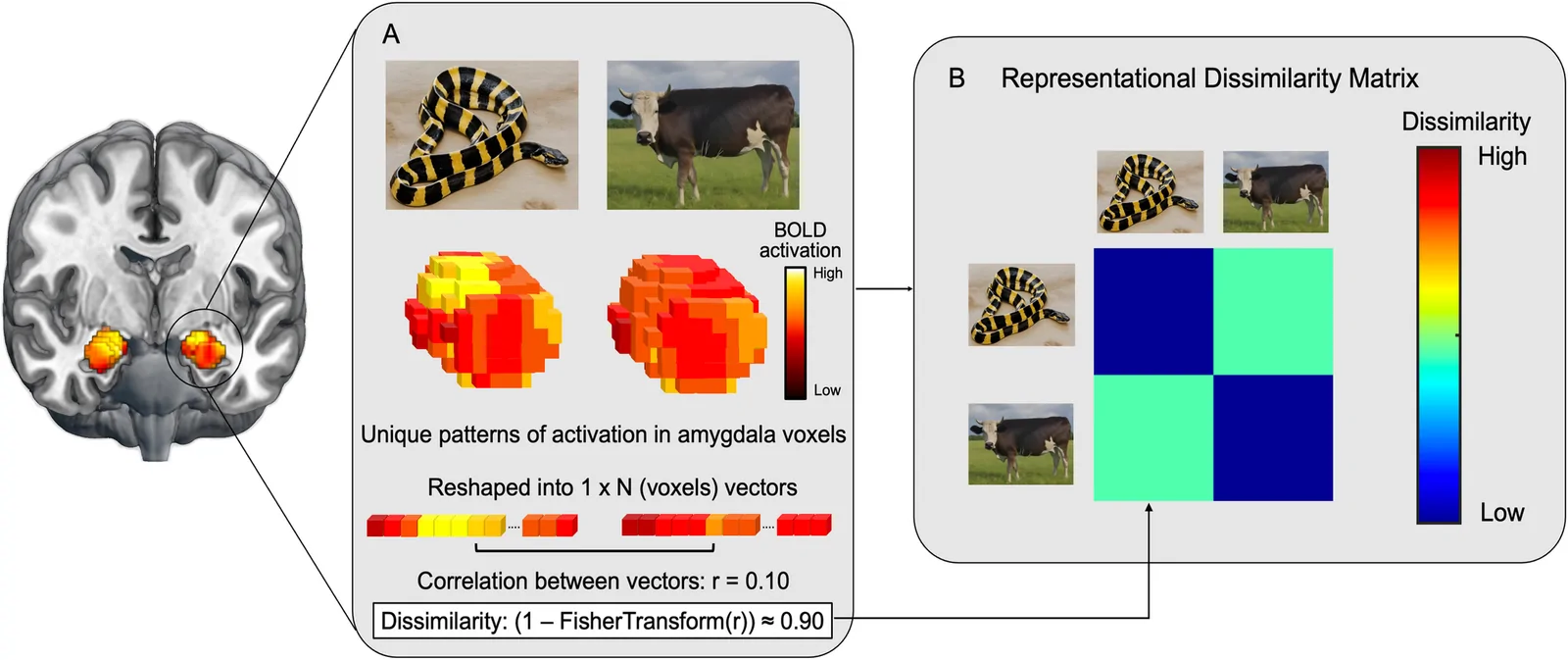
Representational similarity analysis (RSA): An RSA was used to measure the similarity of the neural patterns in the amygdala response to viewing negative compared to neutral images, with less similarity indicating greater differentiation between negative and neutral images. (The correlation and dissimilarity values in this figure are just examples.)

An RSA approach was used to test whether the similarity of the neural patterns in the amygdala in response to viewing negative and neutral images was associated with (i) individual differences in ratings of the images as well as (ii) PET-measured levels of amyloid-β and tau (Fig. 2). Representational pattern similarity measures the degree to which a brain region of interest, such as the amygdala, responds similarly to different types of stimuli using a multivariate pattern taking into account spatial similarity. Here we compare the voxel-wise pattern of neural activation in response to negative compared to neutral images. The RSA MATLAB toolbox (Nili et al., 2014) was used to perform RSA and generate similarity matrices. First, beta maps for each EV were extracted from the GLM constructed in the fMRI analysis. Second, masks of the left and right amygdala regions of interest, extracted from the 2-mm isotropic Harvard-Oxford probabilistic atlas at a probability of 50%, were applied to the beta maps. Then, the left and right amygdala activation pattern to negative and neutral images of each run were reshaped into 1 x N (voxels) vectors. To avoid potential bias generated by temporal collinearity between stimuli in the same run, six between-run negative and neutral image permutations were conducted. An fMRI representational dissimilarity matrix (RDM) was generated by computing the correlation between the activation pattern to negative images and that to neutral images, averaging across permutations, computing the Fisher’s Z transformation, and subtracting the value from 1. The RDM was then inverted to obtain the pattern similarity between viewing negative images and neutral images in the amygdala.

### Univariate and Control Region Comparison Analyses

Comparison analyses are described in the Supplemental Materials using a traditional univariate approach as well as a control region of interest. Univariate analyses were performed to demonstrate the greater sensitivity of the RSA approach. The occipital pole was chosen as a comparison control region after considering the spatial pattern of how amyloid and tau accumulate in the brain since the occipital pole and primary visual cortex are generally preserved until the very late stages of dementia (Grothe et al., 2017; Koscik et al., 2020; LaPoint et al., 2022). Details of the comparison univariate and spatial specificity analysis are provided in Supplementary Material.

### Statistical Analysis

All statistical analyses were computed in RStudio v4.0 + 735. First, regression analyses were conducted to examine whether amygdala pattern similarity to negative and neutral stimuli was associated with participants’ image ratings of valence and arousal and whether those associations differed as a function of biomarker positivity. For amyloid-β positivity, A + was coded as 1, A- was coded as 0. For tau positivity, T + was coded as 1, T- was coded as 0. Then, regression analyses were conducted to examine associations between amygdala pattern similarity to negative and neutral stimuli and biomarker levels (global amyloid-β PiB binding, tau SUVR in the bilateral entorhinal cortex and amygdalae) and whether those associations differed as a function of biomarker positivity. These regression analyses were conducted with and without adjusting for sociodemographic covariates of age, sex (Female, Male), and race (Black/African American, White), and the time lag between fMRI and PET scans.

## Results

### Manipulation Check of Image Ratings

**Fig. 3:**
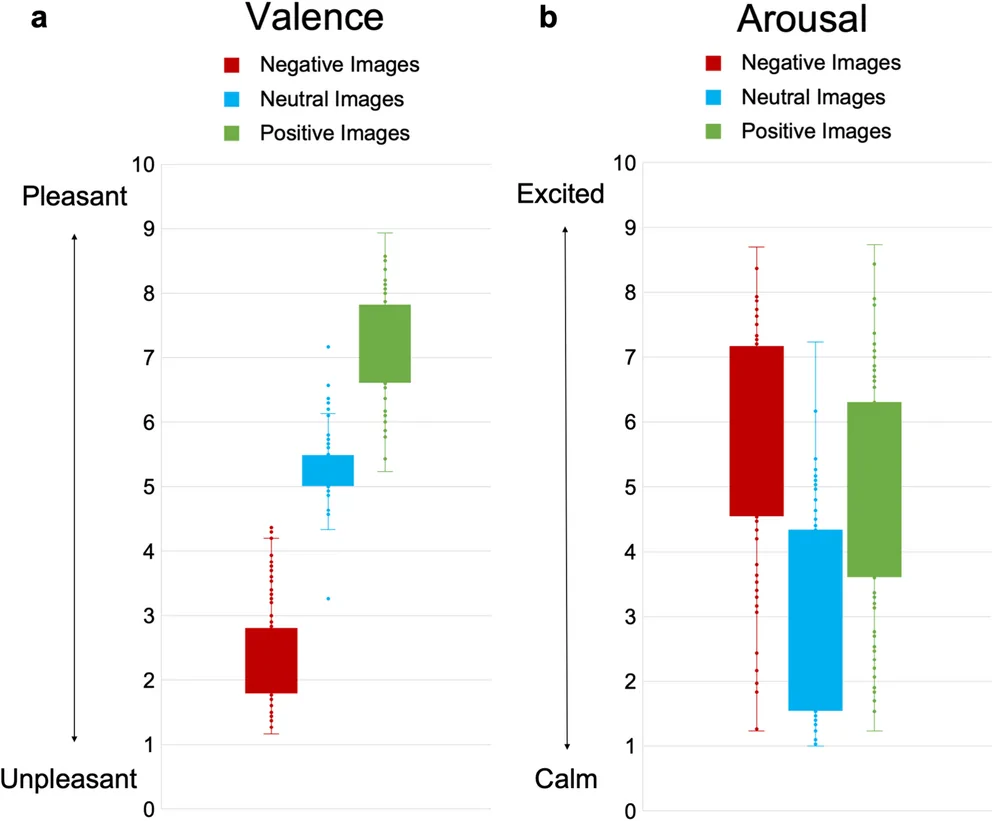
Manipulation Check: Participants rated the images on valence and arousal outside the scanner, and their ratings confirmed the image selection goals. (**a**) Valence ratings. (**b**) Arousal ratings

After completing fMRI scanning, participants reviewed the images and rated them on valence and arousal in terms of how the images made them feel. Their ratings significantly differed between the image categories confirming the image selection goals, with a main effect of image category for valence (Fig. 3; positive: *M *= 7.17, *SD *= 0.78; neutral: *M *= 5.31, *SD *= 0.57; negative: *M *= 2.46, *SD *= 0.81), *F*(2, 240) = 860.52, *p* <.001, and arousal (positive: *M *= 4.92, *SD *= 1.84; neutral: *M *= 2.92, *SD *= 1.53; negative: *M *= 5.76, *SD *= 1.83), *F*(2, 240) = 57.09, *p* <.001.

Paired samples *t*-tests indicated that positive images were rated as more pleasant than neutral images (*t*(80) *=* 24.81, *p* <.001) and negative images were rated as more unpleasant than neutral images (*t*(80) = −27.28, *p* <.001). Both positive and negative images were rated as more arousing than neutral images (positive-neutral: *t*(80) = 13.55, *p* <.001; negative-neutral: *t*(80) = 16.43, *p* <.001), and positive images were rated as less arousing than negative images (*t*(80) = −5.47, *p* <.001).

### Associations Between Amygdala Response Pattern Similarity to Negative and Neutral Images with Image Ratings

**Fig. 4:**
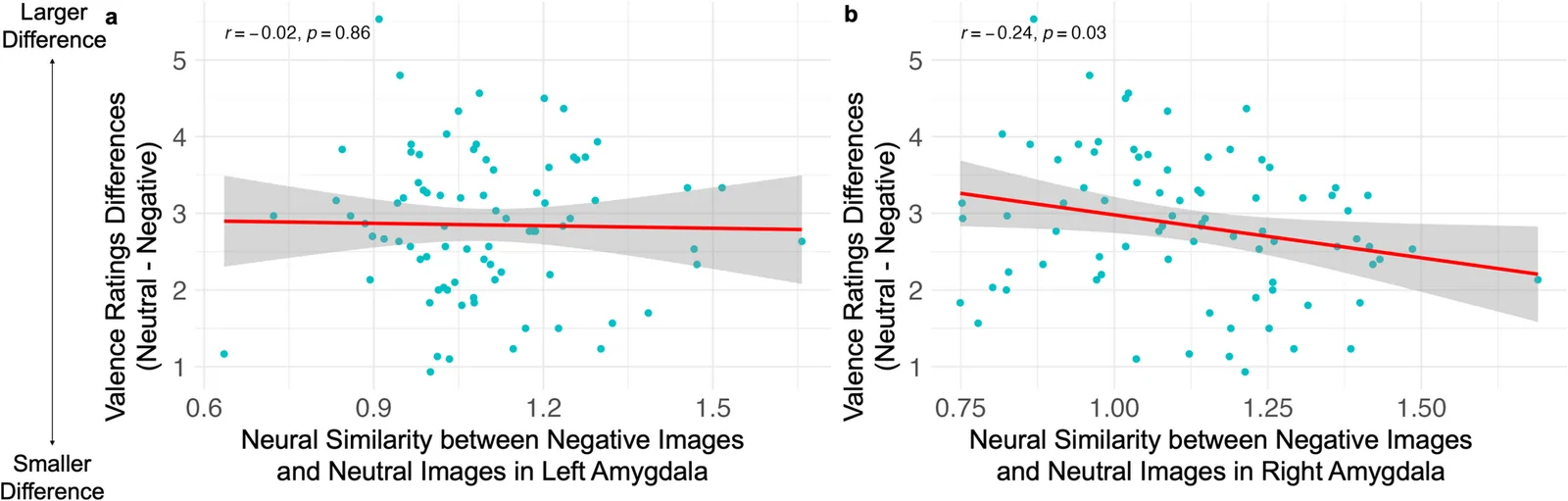
Associations between amygdalar pattern similarity and differences in valence ratings of neutral and negative images. Larger differences in valence ratings of neutral and negative images were associated with less (**b**) right amygdala similarity between negative and neutral images, but not (**a**) left amygdala similarity (uncorrected for multiple comparisons)

Associations were examined between amygdala pattern similarity measures between negative and neutral images with image ratings of valence and arousal. Less right amygdala similarity between negative and neutral images was associated with larger differences in valence ratings between negative and neutral images (Fig. 4b; *β* = −1.12, *t*(79) = −2.17, *p* =.033). This effect was significant when adjusting for age, sex, and race, (*β* = −1.08, *t*(76) = −2.08, *p* =.040). In the same model, the interaction between right amygdala similarity and amyloid-β positivity, the main effect of amyloid-β positivity, the interaction between right amygdala similarity and tau positivity, and the main effect of tau positivity, were not significant (*p*s > 0.2), indicating that the association between valence ratings and amygdala pattern similarity was not influenced by amyloid or tau levels/positivity. Right amygdala similarity between negative and neutral images was not associated with negative images’ valence ratings (*β* = 0.55, *t*(79) = 1.22, *p* =.227). The interaction between right amygdala similarity and amyloid-β positivity, the main effect of amyloid-β positivity, the interaction between right amygdala similarity and tau positivity, and the main effect of tau positivity were also not significant (*p*s > 0.3). Left amygdala similarity between negative and neutral images was not associated with differences in neutral and negative images’ valence ratings (Fig. 4a; *β* = −0.11, *t*(79) = −0.18, *p* =.860). The interaction between left amygdala similarity and amyloid-β positivity, the main effect of amyloid-β positivity, the interaction between left amygdala similarity and tau positivity, and the main effect of tau positivity were also not significant (*p*s > 0.2). Left amygdala similarity between negative images and neutral images was not associated with the valence ratings of negative images (*β* = −0.28, *t*(79) = −0.54, *p* =.588). The interaction between left amygdala similarity and amyloid-β positivity, the main effect of amyloid-β positivity, the interaction between left amygdala similarity and tau positivity, and the main effect of tau positivity were also not significant (*p*s > 0.3). In summary, less right amygdala similarity between negative and neutral images was associated with larger differences in valence ratings between negative and neutral images, and this association was not impacted by either amyloid or tau positivity. However, when adjusted for multiple comparisons, these associations no longer met significance thresholds with a strict Bonferroni correction taking into account the multiple comparisons.

**Fig. 5:**
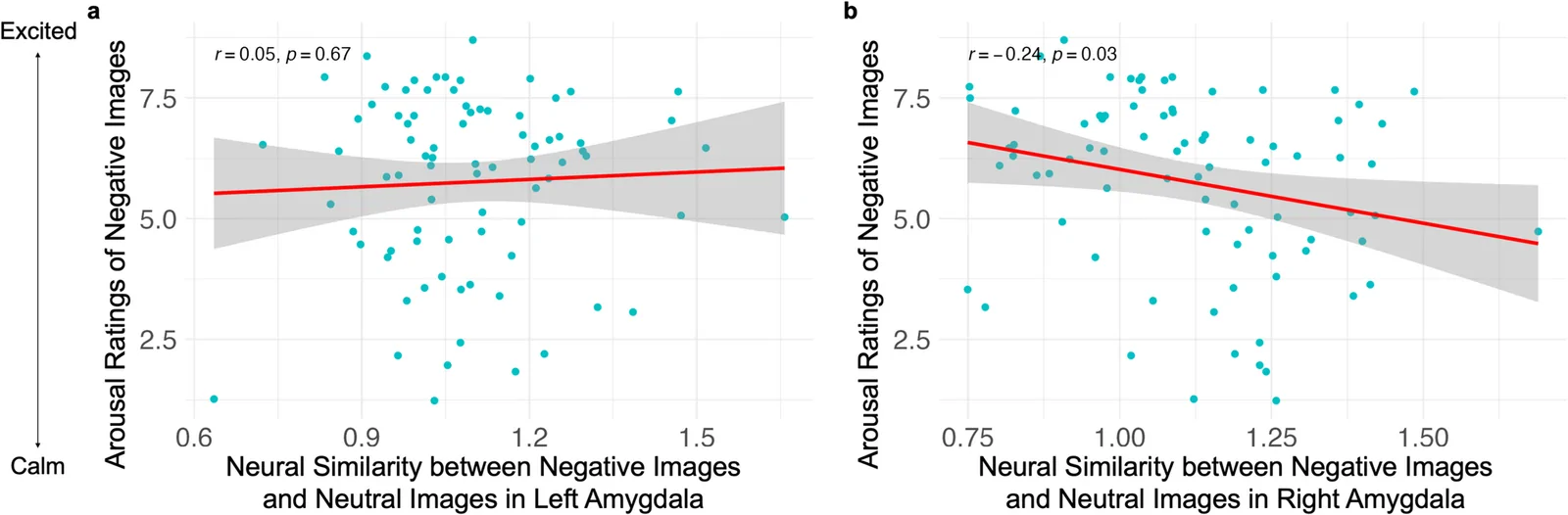
Associations between amygdalar pattern similarity and arousal ratings of negative images. Higher arousal ratings of negative images were associated with less (**b**) right amygdala similarity between negative and neutral images, but not (**a**) left amygdala similarity (uncorrected for multiple comparisons)

Less right amygdala similarity between negative and neutral images was also significantly associated with higher arousal ratings of negative images (Fig. 5b; *β* = −2.23, *t*(79) = −2.22, *p* =.029). This effect was still significant when adjusting for age, sex, and race (*β* = −2.03, *t*(76) = −2.15, *p* =.035). The interaction between right amygdala similarity and amyloid-β positivity, the main effect of amyloid-β positivity, the interaction between right amygdala similarity and tau positivity, and the main effect of tau positivity were not significant (*p*s > 0.5), indicating that the association between arousal ratings and amygdala pattern similarity was not influenced by amyloid or tau levels/positivity. Right amygdala similarity between negative and neutral images was not associated with differences between negative and neutral images’ arousal ratings (*β* = −1.07, *t*(79) = −1.23, *p* =.224). The interaction between right amygdala similarity and amyloid-β positivity, main effect of amyloid-β positivity, the interaction between right amygdala similarity and tau positivity, and the main effect of tau positivity were also not significant (*p*s > 0.2). Left amygdala similarity between negative and neutral images was not associated with negative images’ arousal ratings (Fig. 5a; *β* = 0.51, *t*(79) = 0.43, *p* =.667). The interaction between left amygdala similarity and amyloid-β positivity, main effect of amyloid-β positivity, the interaction between left amygdala similarity and tau positivity, and the main effect of tau positivity were also not significant (*p*s > 0.3). Left amygdala similarity between negative and neutral images was not significantly associated with differences in negative and neutral images’ arousal ratings (*β* = 1.50, *t*(79) = 1.51, *p* =.135). The interaction between left amygdala similarity and amyloid-β positivity, the main effect of amyloid-β positivity, the interaction between left amygdala similarity and tau positivity, and the main effect of tau positivity were also not significant (*p*s > 0.1). Neither left nor right amygdalar similarity between negative and neutral images was significantly associated with arousal ratings of neutral images (*p*s > 0.1). In summary, less right amygdala similarity between negative and neutral images was associated with higher arousal ratings of negative images, and this association was not impacted by either amyloid or tau positivity. However, when adjusted for multiple comparisons, these associations no longer met significance thresholds with a strict Bonferroni correction taking into account the multiple comparisons.

### Associations Between Amygdala Response Pattern Similarity to Negative and Neutral Images with Global Amyloid-β

**Fig. 6:**
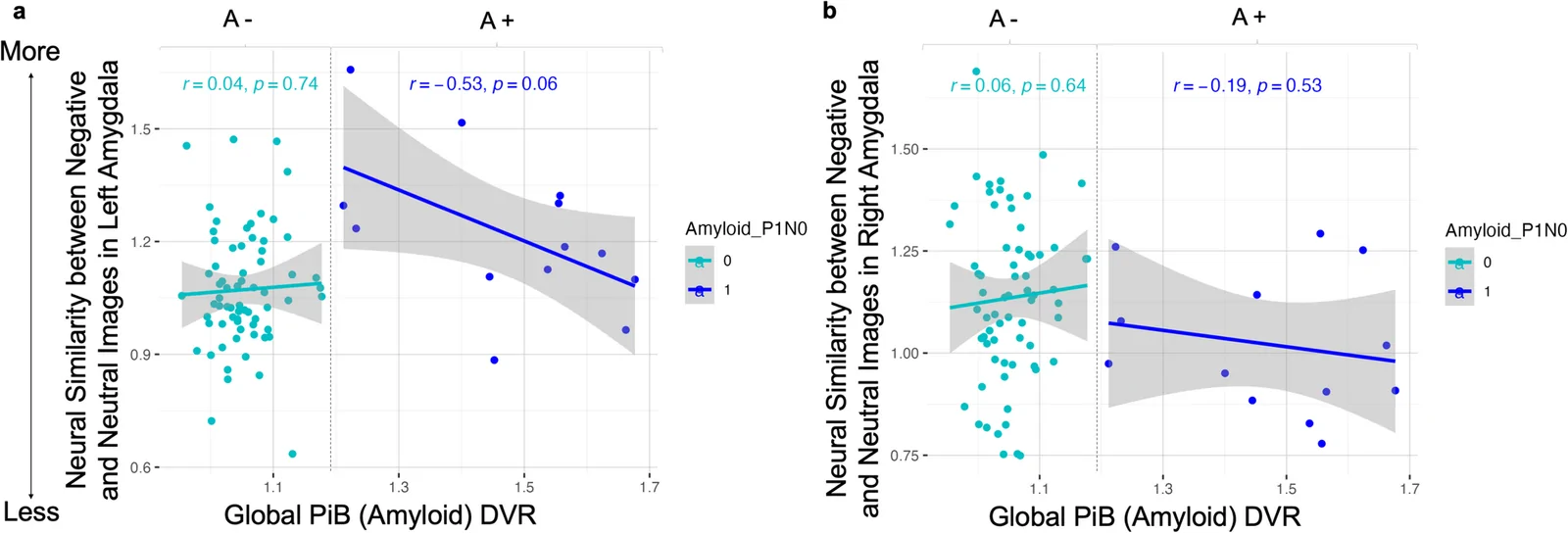
Associations between global amyloid-β DVR and amygdalar pattern similarity measures between negative and neutral images. The interaction between global amyloid-β DVR and amyloid-β positivity was not significant when associated with pattern similarity in the (**a**) left amygdala, or the (**b**) right amygdala. Threshold of A + and A- were set as global amyloid-β DVR = 1.19 indicated by the dashed line. DVR = distribution volume ratio

There was no significant interaction between global amyloid-β PiB binding and amyloid positivity on similarity in the left or right amygdala’s pattern of reactivity (*p*s > 0.1). Global amyloid-β PiB binding was not significantly associated with similarity in the left amygdala’s (Fig. 6a; *β* = 0.20, *t*(79) = 1.81, *p* =.074) or the right amygdala’s pattern of reactivity (Fig. 6b; *β* = −0.23, *t*(79) = −1.81, *p* =.074) between negative and neutral stimuli. There was a significant main effect of amyloid-β positivity on similarity in the left amygdala’s pattern of reactivity between negative and neutral images (*β* = 1.29, *t*(77) = 2.12, *p* =.038), such that the A+ group had on average greater similarity between their reactivity overall than the A- group. However, one outlier, exceeding three standard deviations from the mean value, existed in left amygdala’s pattern similarity between negative and neutral images and was included in the results reported. When excluding the outlier from analyses, amyloid-β positivity no longer reached significance in the association with left amygdala’s pattern similarity between negative and neutral images (interaction: *β* = −0.53, *t*(76) = −1.03, *p* =.308; amyloid-β positivity: *β* = 0.85, *t*(76) = 1.32, *p* =.192; amyloid-β level: *β* = 0.14, *t*(76) = 0.33, *p* =.741). The main effect of amyloid-β positivity on similarity in the right amygdala’s pattern of reactivity was also not significant (*β* = 0.44, *t*(77) = 0.59, *p* =.555). In summary, no significant effects of global amyloid-β PiB binding and amyloid positivity were found on similarity in the left or right amygdala’s pattern of reactivity.

### Associations Between Amygdala Response Pattern Similarity to Negative and Neutral Images with Tau SUVR

**Fig. 7:**
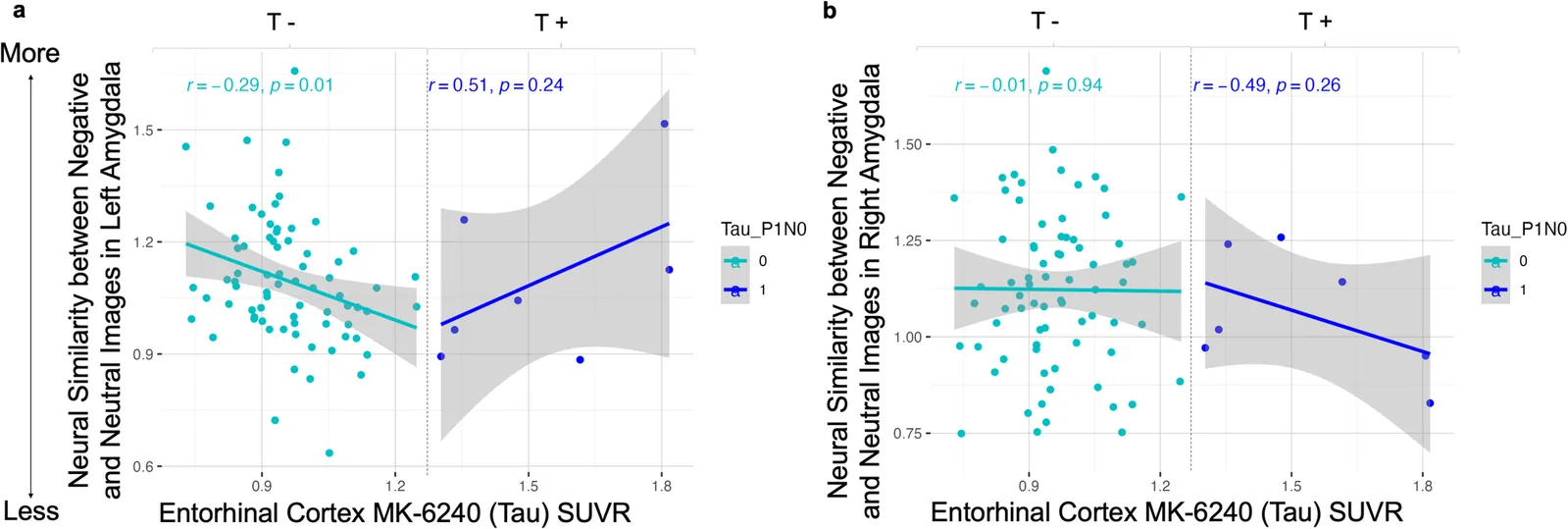
Associations between tau SUVR in the entorhinal cortex and amygdalar pattern similarity measures between negative and neutral images. The interaction between tau SUVR in the entorhinal cortex and tau positivity was significant when associated with pattern similarity in the (**a**) left amygdala, but not the (**b**) right amygdala. Greater tau SUVR in the entorhinal cortex was significantly associated with lower similarity in the left amygdala between negative and neutral stimuli in the T- group, but not in the T+ group (Bonferroni corrected). Threshold of T + and T- were set as entorhinal MK-6240 SUVR = 1.27 indicated by the dashed line. SUVR = standard uptake value ratio

Associations were examined between the amygdala response pattern similarity to negative and neutral images with tau SUVR in the bilateral entorhinal cortex and in the amygdalae. The interaction between tau SUVR in the entorhinal cortex and tau positivity was significant (Fig. 7a; *β* = 0.96, *t*(77) = 2.68, *p* =.009, *Bonferroni adjusted p* =.018), and the main effect of tau SUVR in the entorhinal cortex was significant (*β* = −0.43, *t*(77) = −2.47, *p* =.016, *Bonferroni adjusted p* =.031), such that greater tau SUVR in the entorhinal cortex was associated with lower similarity in the left amygdala’s reactivity between negative and neutral stimuli in the T- group, but not in the T+ group. The main effect of tau positivity was significant (*β* = −1.22, *t*(77) = −2.38, *p* =.020, *Bonferroni adjusted p* =.039), such that the T- group had on average greater similarity between their reactivity overall than the T+ group. Tests of the two a priori hypotheses using Bonferroni-corrected comparisons showed that these regressions were still statistically significant when regarding both left and right amygdala pattern similarity measures. These effects were still significant after adjusting for age, sex, race, and time lag (interaction: *β* = 0.96, *t*(73) = 2.61, *p* =.011; tau level: *β* = −0.42, *t*(73) = −2.29, *p* =.025; tau positivity: *β* = −1.22, *t*(73) = −2.33, *p* =.023). One outlier existed in left amygdala’s pattern similarity between negative and neutral images and was included in the results reported. When excluding the outlier from analyses and adjusting for covariates, these effects were still significant (interaction: *β* = 0.95, *t*(72) = 2.81, *p* =.006; tau level: *β* = −0.41, *t*(72) = −2.38, *p* =.020; tau positivity: *β* = −1.21, *t*(72) = −2.52, *p* =.014). The effect of tau SUVR in the entorhinal cortex on similarity in the left amygdala’s reactivity between negative and neutral stimuli was not significant in the model with no interaction (*β* = −0.07, *t*(79) = −0.75, *p* =.459). The interaction between tau in the entorhinal cortex and tau positivity on right amygdala’s pattern of reactivity, the main effect of tau in the entorhinal cortex, and the main effect of tau positivity were not significant (Fig. 7b; *p*s > 0.4). In summary, less left amygdala similarity between negative and neutral images was associated with higher tau SUVR in the entorhinal cortex in the T-group and this finding was still significant after Bonferroni correction.

**Fig. 8:**
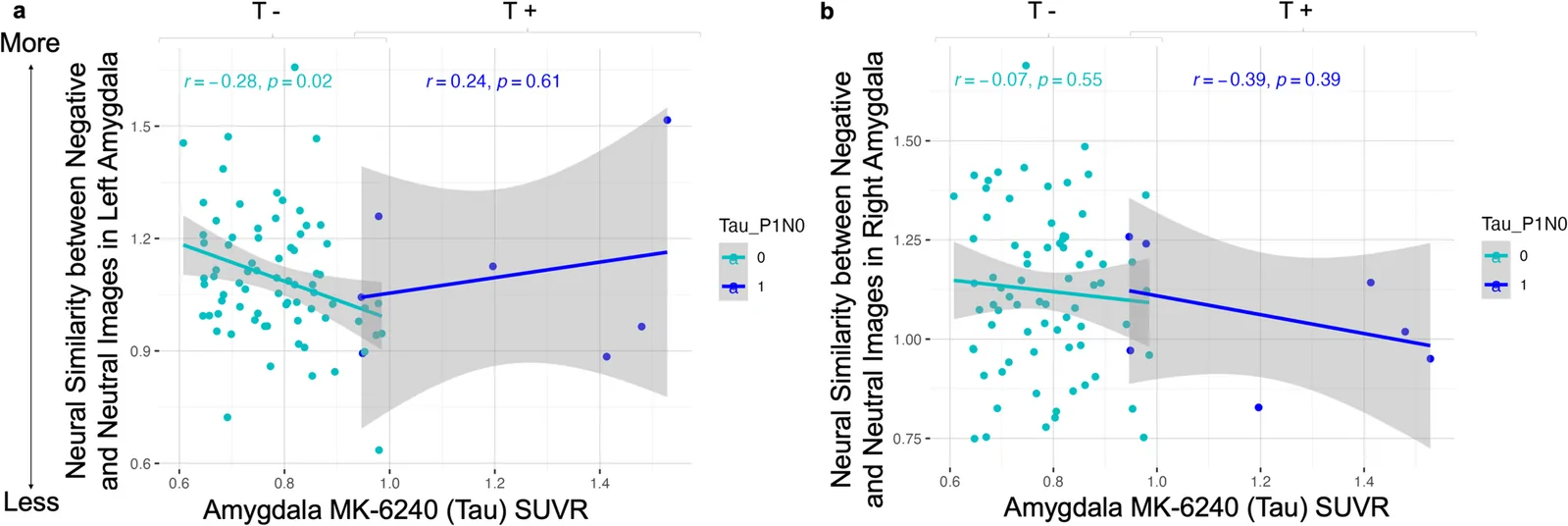
Association between tau SUVR in the amygdalae and amygdalar pattern similarity measures between negative and neutral images. The interaction between tau SUVR in the amygdalae and tau positivity was significant when associated with pattern similarity in the (**a**) left amygdala, but not the (**b**) right amygdala. Greater tau SUVR in the amygdalae were significantly associated with lower similarity in the left amygdala between negative and neutral stimuli in the T- group (Bonferroni corrected). Threshold of T + and T- were set as entorhinal MK-6240 SUVR = 1.27 indicated by the dashed line. SUVR = standard uptake value ratio

The interaction between tau SUVR in the amygdalae and tau positivity was significant but only at trend after Bonferroni correction (Fig. 8a; *β* = 0.71, *t*(77) = 2.09, *p* =.039, *Bonferroni adjusted p* =.079). The main effect of tau SUVR in the amygdalae was significant (*β* = −0.50, *t*(77) = −2.41, *p* =.018, *Bonferroni adjusted p* =.036), such that greater tau SUVR in the amygdalae was associated with less pattern similarity in the left amygdala to negative and neutral images in the T- group, but not in the T+ group. The main effect of tau positivity was not significant (*β* = −0.64, *t*(77) = −1.74, *p* =.086). The interaction effect was no longer significant after adjusting for age, sex, race, and time lag but remained at trend, (interaction: *β* = 0.69, *t*(73) = 1.96, *p* =.054; tau level: *β* = −0.49, *t*(73) = −2.22, *p* =.030; tau positivity: *β* = −0.62, *t*(73) = −1.62, *p* =.111). One outlier existed in the left amygdala’s pattern similarity between negative and neutral images and was included in the results reported. When excluding the outlier from analyses and adjusting for covariates, the interaction effect and the main effect of tau SUVR in the amygdalae were still significant (interaction: *β* = 0.69, *t*(72) = 2.13, *p* =.037; tau level: *β* = −0.49, *t*(72) = −2.38, *p* =.020; tau positivity: *β* = −0.62, *t*(72) = −1.75, *p* =.084). The effect of tau SUVR in the amygdalae on similarity in the left amygdala’s reactivity between negative and neutral stimuli was not significant in the model with no interaction (*β* = −0.11, *t*(79) = −0.93, *p* =.353). The interaction effect between tau in the amygdalae and tau positivity on right amygdala’s pattern of reactivity, the main effect of tau in the amygdala, and the main effect of tau positivity were not significant (Fig. 8b; *p*s > 0.2). In summary, less left amygdala similarity between negative and neutral images was associated with higher tau SUVR in the amygdalae in the T- group and this finding was still significant after Bonferroni correction.

### Univariate and Control Region Comparison Analyses

Results of the univariate and control region analyses confirm greater sensitivity with the RSA approach and spatial specificity of the findings such that associations were found between tau levels and amygdalar similarity metrics but not occipital pole similarity metrics. See Supplementary Materials for details.

## Discussion

In a sample of older adults from the Wisconsin Registry for Alzheimer’s Prevention, we found representational pattern similarity in the amygdala to negative compared to neutral images were associated with both the participants’ ratings of the stimuli and their PET-measured tau levels in the entorhinal cortex and amygdalae. Although no significant relations were found with global amyloid levels, less right amygdala pattern similarity between negative and neutral images was associated with larger differences in participants’ valence ratings between negative and neutral images as well as higher arousal ratings of the negative images. Although these associations survived after adjusting for sociodemographics, these associations with participants’ ratings were no longer significant after Bonferroni correction adjusting for multiple comparisons across left and right amygdala and rating metrics. Associations between bilateral entorhinal and amygdalar tau with left amygdala pattern similarity between negative and neutral images differed depending on whether entorhinal tau SUVRs were above or below previously defined threshold of T+ positivity (Betthauser et al., 2020), such that greater tau levels in the entorhinal cortex or amygdalae were associated with less left amygdala pattern similarity between negative and neutral images in participants categorized as T-. Importantly, the association between tau levels in the entorhinal cortex and the amygdala pattern similarity remained significant even after adjusting for sociodemographics, the time lag between the scans, and multiple comparisons across left and right amygdalae. Thus, in summary, we found that fMRI indices of amygdalar differentiation between negative and neutral stimuli were associated with higher tau levels in the entorhinal cortex of a subsample of older adults below PET-defined T+ positivity from the Wisconsin Registry for Alzheimer’s Prevention.

The larger valence difference people perceived between negative and neutral images and the more arousing they found the negative images, the larger their difference in the right amygdala’s pattern of reactivity to those same stimuli. The larger valence difference reflected a greater difference in how participants reported feeling (unhappy compared to happy) in response to the negative and neutral images and greater arousal reflected feeling more excited compared to calm in response to the negative images. Therefore, we interpret less similarity (or larger differentiation) in amygdalar pattern similarity to negative and neutral stimuli as greater emotional reactivity or sensitivity to the negative stimuli if perceived different in valence from neutral stimuli and more arousing. This greater sensitivity to negative, unpleasant, and threatening exposures might underlie some of the emotional abnormalities in the preclinical stage of AD and Related Dementias. In other words, high levels of irritability, anxiety, and depression (Fredericks et al., 2018; Johansson et al., 2022; Tristão-Pereira et al., 2025) and emotion regulation deficits in FTD (Goodkind et al., 2010), may reflect and/or result from greater sensitivity to emotionally provocative, unpleasant, or adverse information, experiences, and situations. These findings are consistent with greater similarity in perceived valence ratings of odors being related to greater similarity in amygdala response patterns (Jin et al., 2015) and previous reports of activation in the right amygdala elicited by unpleasant compared to neutral images positively correlating with arousal ratings (Gerdes, 2010). Our results, found across all participants in the right amygdala, provide support for the link between individual differences in implicit right amygdalar emotional reactivity to negative images and the explicit self-reported perceived valence and arousal of those same stimuli regardless of amyloid or tau levels/positivity. However, it is important to note that these associations with the ratings, although informative and still significant after adjusting for sociodemographics, do not survive Bonferroni correction when adjusted for multiple comparisons in our limited sample.

Nonetheless, we think these significant uncorrected associations are informative for interpreting the RSA findings, and believe the associations with different ratings metrics make sense when one considers the nature of the ratings. Amygdalar RSA was associated with the *differenc*e in valence ratings between negative and neutral images, but with negative image arousal ratings themselves (but not for neutral images), likely due to the different distributions and ranges in the ratings for valence vs. arousal. The participants’ valence ratings of negative and neutral images exhibit range restriction, since the images were originally selected based on the normative IAPS ratings to be negative or neutral exemplars. Relationships are easier to detect with greater spread (variance) in ratings. This range restriction, when examined individually within valence sets, is somewhat alleviated by examining the differences between the valences. For example, the ranges of the participants’ valence ratings of both negative images (1.17 ~ 4.37) and the neutral images (3.27 ~ 7.17) are much narrower than the ranges of arousal ratings of negative images (1.23 ~ 8.70) and neutral images (1.00 ~ 7.23). Therefore, the amygdala pattern similarity associations with differences in valence ratings between negative and neutral images, but with arousal ratings of the negative images likely reflect the ability to detect relationships given differences in range. Notably, associations between higher arousal ratings of unpleasant images and stronger activation in the right amygdala have previously been reported in the literature (Berntson et al., 2007; Gerdes, 2010). Moreover, patients with amygdala lesions rated negative stimuli as less arousing than healthy controls, despite their ratings of neutral and positive stimuli being similar (Berntson et al., 2007).

Interaction effects were found between both entorhinal cortex tau levels and tau positivity thresholds and between amygdalar tau levels and tau positivity thresholds with left amygdala pattern similarity between negative and neutral images. Greater entorhinal and amygdalar tau were related to less left amygdala pattern similarity to negative and neutral images in participants categorized as T-. Although the interaction effect of amygdalar tau levels and tau positivity thresholds was not significant after Bonferroni correction, entorhinal and amygdalar tau remain significantly associated with left amygdala pattern similarity to negative and neutral images after Bonferroni correction. These associations may help explain early emotional abnormalities in preclinical AD and other disorders characterized by tauopathies through greater neural differentiation between negative and neutral stimuli and greater sensitivity to the emotional content in the images. The accumulation of amygdalar tau might influence amygdala functioning and emotion processing. This is consistent with a previous study that found greater amygdalar tau levels were associated with greater depressive symptoms in an AD-risk sample (Tristão-Pereira et al., 2025). Tau levels in the temporal lobe, which contains the entorhinal cortex, have previously been shown to track dementia more closely and more strongly predict cognitive impairment than amyloid-β deposition (Brier et al., 2016). Although amyloid accumulation is an important biomarker of AD pathology, amyloid does not always lead to clinical symptoms. Tau pathology may be more closely linked to neurodegeneration and symptoms progression, and recent evidence suggests tau may be a potentially more informative marker of AD (Courade et al., 2025; Ossenkoppele et al., 2016; Therriault et al., 2024; Villemagne et al., 2018) and the FTD (Bodea et al., 2016).

We only found the association between amygdala reactivity and tau levels in participants categorized as T-. Above the tau positivity threshold, excessive tau might lead to neurodegeneration and altered amygdalar responding disrupting the association. However, because our study is cross-sectional and critically limited by the small number of participants who were categorized as T+, we simply cannot make any conclusions regarding the T+ group. Additional work is needed examining amygdalar responses to emotional stimuli in both A + and T+ participants, longitudinally tracking disease progression with their emotional responses. In this study, the associations between tau levels and amygdala reactivity were only significant in the left amygdala, while valence and arousal ratings were associated with right amygdala reactivity. Also noteworthy, a similar pattern was observed in the associations between tau levels and right amygdala reactivity in participants categorized as T-, but the association did not reach statistical significance. Because tau levels were assessed bilaterally in our study, we also cannot draw conclusions about differential impairment from left vs. right amygdalar tau pathology. Replication is needed in larger samples to determine if the left and right amygdala are differentially involved in emotional reactivity processes with tau pathology.

Surprisingly, no significant association was found between global amyloid-β level and amygdalar representational similarity to negative and neutral images. Although we initially found a significant main effect of amyloid-β positivity on left amygdalar pattern similarity between negative and neutral images, this effect was no longer significant after removing an outlier datapoint. Since only 13 participants were in the A+ group and we were considerably under-powered, this effect also deserves future investigation in larger samples. A possible reason why significant associations were found between amygdalar pattern similarity with entorhinal and amygdalar tau levels below positivity levels, but not for global amyloid-β, might be that amyloid levels were based on *global* rather than *regional* estimates given the inherent patterns of how these proteins differentially accumulate in the brain. Amyloid-β pathology typically spreads more diffusely across distant brain regions, whereas tau accumulation shows a hierarchical pattern following Braak staging and tends to be more regionally focused starting in the temporal lobe (Braak & Braak, 1991; Grothe et al., 2017; Guzmán-Vélez et al., 2022). The temporal regional focus of tau pathology may impact emotional processes to a greater degree than amyloid, as suggested by our findings that entorhinal and amygdalar tau levels were more strongly associated with amygdalar reactivity than global amyloid-β level. These findings suggest older adults’ amygdalar reactivity to unpleasant events, situations, and experiences in their daily lives might differ according to tau accumulation impacting their subsequent emotional responses. However, additional work is needed to extend our findings to daily experiences to investigate whether accumulating tau contributes vulnerability and/or greater sensitivity to unpleasantness, threat, and adversity in daily life.

For comparisons with the RSA approach, we performed a more traditional univariate analysis with difference scores between negative and neutral images and found they were not related to amyloid-β or tau levels, suggesting that the RSA approach may be more sensitive to individual differences in amygdalar patterns of reactivity to emotional stimuli. Superior sensitivity of RSA compared to univariate analyses has been previously reported (Chavez & Heatherton, 2015; Davis et al., 2021; Puccetti et al., 2021). The associations between amygdalar reactivity and tau levels were also spatially specific to the amygdala when compared with the occipital pole control ROI.

Notably, our study has considerable limitations. First, the sample sizes of A + and T+ group were small with only 13 participants in the A+ group and 7 participants in the T+ group and some overlap between groups. Significant findings between amygdala RSA and tau SUVR were all within the “subthreshold” range, which may be more susceptible to stochastic measurement and image processing errors. Therefore, our preliminary findings need replication. Hopefully future studies in larger participant samples of both A+/- and T+/- participants can replicate and extend the results. Secondly, this study focused only on amygdalar reactivity to negative compared to neutral stimuli. Future studies need to examine whether the individual differences in neural responses to positive vs. neutral stimuli are similarly related to pathological protein accumulation in older adults and include other regions of interest important for affective processes. Given the often-reported complaints of negative emotional symptoms in AD trajectories, our goal was to first examine neural measures of negative emotion in the WRAP sample with a focus on the amygdala, but deficits in positive emotionality may exist and deserve future investigation. Thirdly, although AD has been associated with alterations in affective processing and disruption of emotion regulation (Dafsari & Jessen, 2020; Harerimana et al., 2022; Korthauer et al., 2018; Liu et al., 2024; Ng et al., 2017; Turnbull et al., 2026; Wang et al., 2019), other neurodegenerative disorders such as byFTD (Piguet & Hodges, 2013) and Lewy Body dementia (Segers et al., 2020) also experience emotional abnormalities which may differ from AD. The progression of participants in this study to AD, FTD, other Related Dementias, or no progression remains unknown. FTD is at least partially attributed to tauopathies, these findings may have relevance to other neurodegenerative conditions beyond AD.

In conclusion, this study examined associations between representational pattern similarity in the amygdala’s reactivity to negative and neutral stimuli, ratings of valence and arousal of the stimuli, and amyloid-β and tau levels in the brain in older adults without dementia from the Wisconsin Registry for Alzheimer’s Prevention. Larger differences between valence ratings of neutral and negative images and higher arousal ratings of negative images were associated with less right amygdala similarity between negative and neutral images (uncorrected for multiple comparisons). Greater bilateral entorhinal and amygdalar tau levels were associated with less left amygdala pattern similarity between negative and neutral images in individuals whose tau SUVR was below the positivity threshold. These preliminary findings suggest accumulating tau in the brain is associated with more amygdalar differentiation between negative and neutral stimuli indicative of a greater amygdalar sensitivity to negative affective information. More research is needed to replicate and extend the findings in larger samples. Nonetheless, our results suggest a potential neural basis for the often-reported negative emotionality with higher levels of anxiety, depression, and apathy that often accompanies early stages on the trajectory to dementia in AD.

## Supplementary Information

Below is the link to the electronic supplementary material.

Supplementary Material 1 (DOCX 128 KB)

## Additional Information

### Data Availability

Data are available upon request through the Wisconsin Registry for Alzheimer’s Prevention at the following link: https://wrap.wisc.edu/data-requests-2/.

### Code availability

The code used for analyses and plots are publicly available on the Open Science Framework at the following link: https://osf.io/qswk6/files/osfstorage.
