# Supplementary material for: Amygdalar Pattern Similarity to Negative and Neutral Images, Ratings of the Images, and PET-Measured Amyloid and Tau Levels in Older Adults Without Dementia
Source: Affect Sci. 2026 Aug 11;7(3):483–97. doi: 10.1007/s42761-026-00394-5 (PMC13582699; doi:10.1007/s42761-026-00394-5)
Supplement: Supplementary file 1 [file 42761_2026_394_MOESM1_ESM.docx]

**Supplement:** Amygdalar Pattern Similarity to Negative and Neutral Images, Ratings of the Images, and PET-measured Amyloid and Tau Levels in Older Adults without Dementia

**Method**

**Inclusion and Exclusion Criteria**

Main Inclusion Criteria:

- Participation in the Wisconsin Registry for Alzheimer’s Prevention (WRAP) study and an ancillary study which included MR and PET imaging whose inclusion criteria included: An active participant in the main WRAP study.
- Age: Between 50 and 80 at entry (thus making age for this protocol between 50-85).
- No dementia:  At risk participants do not exhibit dementia criteria at enrollment into this study.
- Availability of WRAP assessments: APOE status and valid cognitive testing available from the main WRAP study.
- Good general health with no conditions/medications affecting cognition or imaging.
- Commitment and ability to tolerate imaging procedures and optional lumbar puncture.
- Willingness to participate, sign consent form, and comply with the protocol.

Main Exclusion Criteria:

- Significant neurologic disease: Any significant neurologic disease, such as Parkinson’s disease, stroke, Huntington’s disease, normal pressure hydrocephalus, brain tumor, progressive supranuclear palsy, subdural hematoma, multiple sclerosis, seizure disorder, or history of significant head trauma (30 min or more of loss of consciousness) followed by persistent neurologic deficits or known structural brain abnormalities.
- MRI contra-indications: Presence of non-compatible pacemakers/defibrillators, aneurysm clips, artificial heart valves, ear implants, metal fragments, or foreign objects in the eyes, skin, body. Medical records, CT or x-ray may be used to establish suitability for MRI.
- Neuroimaging: Abnormal MRI performed at screening with evidence of infection, infarction, or other focal lesions. This is based on the radiologist’s judgment of current and/or prior MRIs.
- Pregnancy: Participant is not pregnant at the time of the PET and MRI imaging exams. Urine pregnancy tests may be conducted with pre-menopausal women.
- Alcohol/Substance: History of alcohol/substance dependence since joining the source cohort.
- Psychiatric disorders: Current significant Axis I DSM-V major disorders including but not limited to Major depression since joining the cohort, current or history of bipolar I disorder since joining the cohort, schizophrenia spectrum disorders, or history of significant learning disability.
- Significant medical illness: Any significant current or past systemic illness or unstable medical condition occurring during WRAP participation that could affect cognition, or imaging or LP procedures or results. Examples include malignant cancer, chemotherapy, poorly controlled hypertension, heart failure, or renal insufficiency.
- Cognitive Capacity: Participants who do not have the cognitive competence and legal capacity to make informed medical decisions are excluded at entry.
- Vulnerable subjects: Subjects who lack capacity, prisoners, non-English speaking, or otherwise unable to read consent materials are also excluded.
- Excluded Medications: Current use of antipsychotic medications. Warfarin or similar medication (if consenting to LP).

**Univariate Analysis and Spatial Specificity**

Following previous examples of studies using the RSA approach including comparative univariate analyses (e.g. Puccetti et al. 2021), we also performed comparison univariate analyses to demonstrate the greater sensitivity of the RSA approach. Standard univariate analyses compare averaged activities in response to different conditions such as the amygdala’s average activity in response to negative versus neutral stimuli. Because univariate analysis averages activity across all voxels in the brain region of interest, fine-grained, spatial activity patterns are lost. In the univariate analysis, the mean time course of left and right amygdala in response to negative and neutral images was extracted for each participant using fsl. Anatomical masks of left and right amygdala extracted from the 2-mm isotropic Harvard-Oxford probabilistic atlas at a probability of 50% was applied to the COPE images of negative image viewing and neutral image viewing. Univariate difference scores were absolute values of the differences between negative image viewing and neutral image viewing (Puccetti et al., 2021). These scores were used in the subsequent analysis to examine its relationship with the amyloid and tau biomarker levels.

In order to examine whether there is a spatially specific relationship between the amygdala’s reactivity to negative and neutral images and amyloid-β PiB binding and tau SUVR, RSA was also applied to a control region of the occipital pole which is responsible for visual processing (Odimayo et al., 2024; Rehman & Al Khalili, 2024). The occipital pole was selected as the control region after considering the spatial pattern of how amyloid and tau typically accumulate in the brain with the occipital pole (aka primary visual cortex) being generally preserved until the very late stages of dementia (Grothe et al., 2017; Koscik et al., 2020; LaPoint et al., 2022).

**Results**

**Univariate Analysis and Spatial Specificity**

Previous studies have demonstrated greater sensitivity of the RSA approach, which takes advantage of the multivariate nature of fMRI data compared to univariate analyses (Chavez & Heatherton, 2015; Davis et al., 2021; Pillet et al., 2020; Puccetti et al., 2021). In order to demonstrate the superior RSA sensitivity compared to univariate analyses in our project, univariate analyses were conducted. Global amyloid-β level was not significantly associated with univariate difference scores between negative and neutral images from right amygdala (*β* = -3.78, *t*(79) = -0.59, *p* = .554). Although global amyloid-β level was associated with univariate difference scores between negative and neutral images from the left amygdala (*β* = -16.70, *t*(79) = -2.24, *p* = .028), this association was no longer significant after adjusting for covariates (*β* = -14.10, *t*(75) = -1.85, *p* = .069). Interaction effects between global amyloid-β level and amyloid-β positivity, and main effect of amyloid-β positivity were not significant (*p*s > .2).

Similarly, univariate difference scores between negative and neutral image from the left amygdala were not significantly associated with tau SUVR in the entorhinal cortex (*β* = -8.54, *t*(79) = -1.32, *p* = .192) or in the amygdalae (*β* = -14.65, *t*(79) = -1.89, *p* = .062). Univariate difference scores between negative and neutral images from the right amygdala were also not associated with tau SUVR in the entorhinal cortex (*β* = -1.86, *t*(79) = -0.34, *p* = .733) or in the amygdalae (*β* = -2.09, *t*(79) = -0.32, *p* = .751). Interaction effects between tau SUVR in the entorhinal cortex and tau positivity, interaction effects between tau SUVR in the amygdalae and tau positivity, and the main effect of tau positivity were all not significant (*p*s > .6).

To examine whether the relationship between the amygdala’s reactivity to negative and neutral stimuli and tau levels were spatially specific to the amygdala, the control region of the bilateral occipital pole was used in a control representational similarity analysis. The interaction effect between global amyloid-β levels and amyloid-β positivity on the similarity in occipital pole’s reactivity between negative and neutral stimuli was significant (*β* = -2.26, *t*(77) = -2.14, *p* = .035). Global amyloid-β level was not associated with the occipital pole’s pattern similarity between negative and neutral stimuli (*β* = -0.37, *t*(79) = -1.60, *p* = .113). There was a significant main effect of amyloid-β positivity on the occipital pole’s similarity between negative and neutral stimuli (*β* = 3.19, *t*(77) = 2.50, *p* = .014). One outlier existed in the occipital pole’s pattern similarity between negative and neutral images and was included in the results reported. When excluding the outlier from analyses, the interaction effect and the main effect of amyloid-β positivity were no longer significant (interaction: *β* = -1.55, *t*(76) = -1.51, *p* = .135; amyloid-β positivity: *β* = 2.23, *t*(76) = 1.79, *p* = .078). Tau SUVR in the entorhinal cortex was not significantly associated with the similarity in occipital pole’s reactivity between negative and neutral stimuli (*β* = 0.03, *t*(79) = 0.16, *p* = .872). Tau SUVR in the amygdalae was not associated with the similarity in the occipital pole’s reactivity between negative and neutral stimuli (*β* = 0.04, *t*(79) = 0.18, *p* = .858). Interaction effects between tau SUVR in the entorhinal cortex and tau positivity, interaction effects between tau SUVR in the amygdalae and tau positivity, and main effect of tau positivity were not significant (*p*s > .5). In summary, RSA of the occipital pole control region of interest’s pattern of response to negative compared to neutral images did not exhibit any significant relations with amyloid or tau levels or positivity.

**References**

Chavez, R. S., & Heatherton, T. F. (2015). Representational Similarity of Social and Valence Information in the Medial pFC. *Journal of Cognitive Neuroscience*, *27*(1), 73–82. https://doi.org/10.1162/jocn_a_00697

Davis, S. W., Geib, B. R., Wing, E. A., Wang, W.-C., Hovhannisyan, M., Monge, Z. A., & Cabeza, R. (2021). Visual and Semantic Representations Predict Subsequent Memory in Perceptual and Conceptual Memory Tests. *Cerebral Cortex*, *31*(2), 974–992. https://doi.org/10.1093/cercor/bhaa269

Grothe, M. J., Barthel, H., Sepulcre, J., Dyrba, M., Sabri, O., Teipel, S. J., For the Alzheimer’s Disease Neuroimaging Initiative, Alzheimer’s Disease Neuroimaging Initiative, Weiner, M., Aisen, P., Weiner, M., Aisen, P., Petersen, R., Jack, C. R., Jagust, W., Trojanowki, J. Q., Toga, A. W., Beckett, L., Green, R. C., … Raj, B. A. (2017). In vivo staging of regional amyloid deposition. *Neurology*, *89*(20), 2031–2038. https://doi.org/10.1212/WNL.0000000000004643

Koscik, R. L., Betthauser, T. J., Jonaitis, E. M., Allison, S. L., Clark, L. R., Hermann, B. P., Cody, K. A., Engle, J. W., Barnhart, T. E., Stone, C. K., Chin, N. A., Carlsson, C. M., Asthana, S., Christian, B. T., & Johnson, S. C. (2020). Amyloid Duration is Associated with Preclinical Cognitive Decline and Tau PET. *Alzheimer’s & Dementia: Diagnosis, Assessment & Disease Monitoring*, *12*(1). https://doi.org/10.1002/dad2.12007

LaPoint, M. R., Baker, S. L., Landau, S. M., Harrison, T. M., & Jagust, W. J. (2022). Rates of β-Amyloid Deposition Indicate Widespread Simultaneous Accumulation throughout the Brain. *Neurobiology of Aging*, *115*, 1–11. https://doi.org/10.1016/j.neurobiolaging.2022.03.005

Odimayo, S., Olisah, C. C., & Mohammed, K. (2024). Structure Focused Neurodegeneration Convolutional Neural Network for Modelling and Classification of Alzheimer’s Disease. *Scientific Reports*, *14*(1), 15270. https://doi.org/10.1038/s41598-024-60611-8

Pillet, I., Op De Beeck, H., & Lee Masson, H. (2020). A Comparison of Functional Networks Derived from Representational Similarity, Functional Connectivity, and Univariate Analyses. *Frontiers in Neuroscience*, *13*, 1348. https://doi.org/10.3389/fnins.2019.01348

Puccetti, N. A., Schaefer, S. M., Van Reekum, C. M., Ong, A. D., Almeida, D. M., Ryff, C. D., Davidson, R. J., & Heller, A. S. (2021). Linking Amygdala Persistence to Real-World Emotional Experience and Psychological Well-Being. *The Journal of Neuroscience*, 41(16), 3721-3730. https://doi.org/10.1523/JNEUROSCI.1637-20.2021

Rehman, A., & Al Khalili, Y. (2024). Neuroanatomy, Occipital Lobe. In *StatPearls*. StatPearls Publishing. http://www.ncbi.nlm.nih.gov/books/NBK544320/
